# Synthesis and in vitro/in vivo Evaluation of the Antitrypanosomal Activity of 3-Bromoacivicin, a Potent CTP Synthetase Inhibitor

**DOI:** 10.1002/cmdc.201000417

**Published:** 2010-12-22

**Authors:** Paola Conti, Andrea Pinto, Pui E Wong, Louise L Major, Lucia Tamborini, Maria C Iannuzzi, Carlo De Micheli, Michael P Barrett, Terry K Smith

**Affiliations:** a Dipartimento di Scienze Farmaceutiche “P. Pratesi”, Università degli Studi di MilanoVia Mangiagalli 25, 20133 Milano (Italy), Fax: (+39) 02-50319326; b Wellcome Trust Centre of Molecular Parasitology, Institute of Infection, Immunity and Inflammation, College of Medical, Veterinary and Life Sciences, University of GlasgowGlasgow, G12 8TA (Scotland), Fax: (+44) 141-330-4600; c Centre for Biomolecular Sciences, St Andrews UniversitySt Andrews, KY16 9ST (Scotland), Fax: (+44) 1334-462595

**Keywords:** amino acids, CTP synthetase, inhibitors, transferases, trypanosoma

## Abstract

The first convenient synthesis of enantiomerically pure (α*S*,5*S*)-α-amino-3-bromo-4,5-dihydroisoxazol-5-yl acetic acid (3-bromoacivicin) is described. We demonstrate that 3-bromoacivicin is a CTP synthetase inhibitor three times as potent as its 3-chloro analogue, the natural antibiotic acivicin. Because CTP synthetase was suggested to be a potential drug target in African trypanosomes, the in vitro/in vivo antitrypanosomal activity of 3-bromoacivicin was assessed in comparison with acivicin. Beyond expectation, we observed a 12-fold enhancement in the in vitro antitrypanosomal activity, while toxicity against mammalian cells remained unaffected. Despite its good in vitro activity and selectivity, 3-bromoacivicin proved to be trypanostatic and failed to completely eradicate the infection when tested in vivo at its maximum tolerable dose.

## Introduction

Human African trypanosomiasis (HAT) is caused by protozoan parasites of the *Trypanosoma brucei* subgroup.^1^, ^2^ Two subspecies are responsible for causing the disease in humans: *T. b. gambiense* and *T. b. rhodesiense. T. b. gambiense* is endemic in western and central Africa, causing a chronic form of the sickness, whereas *T. b. rhodesiense* is restricted to eastern and southern Africa, causing an acute illness that leads to death within a few weeks of infection. The trypanosomes are transmitted by tsetse flies to their mammalian hosts, where they first establish an infection in the blood and lymph. In the second stage of the disease, the parasites invade the central nervous system (CNS). Chemotherapy is currently the main way to control this disease, as there are no effective vaccines. However, currently available treatments suffer numerous drawbacks including poor efficacy, difficulties to administer, insecure supplies, cost, and an increasing threat of resistance. Therefore, there is a great need for new drugs, and efforts have been made to identify new molecular targets and innovative therapeutic tools.

Cytidine triphosphate synthetase (CTPS), a glutamine amidotransferase (GAT) responsible for the de novo synthesis of CTP, has been proposed and recently genetically validated as a potential drug target for the treatment of HAT.^3^ In fact, despite CTP being essential for cell survival, *T. brucei* has low pools of CTP relative to mammalian cells and completely lacks the ability to salvage cytosine or cytidine.^3^

**Figure 1:**
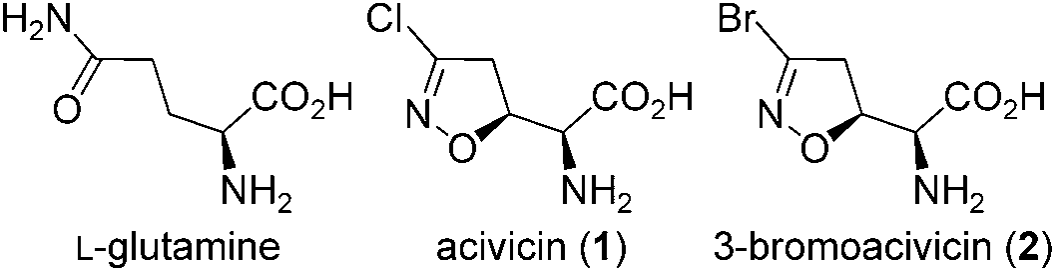
Structure of L-glutamine and target compounds.

Acivicin (Figure 1), an antibiotic isolated from the fermentation broths of *Streptomyces sviceus*, previously tested as an antitumor agent, has long been known to behave as an inhibitor of several GATs including those which play important roles in purine and pyrimidine metabolism.^4^ More recently, its CTPS inhibitory activity has been correlated to the observed antitrypanosomal activity against bloodstream *T. brucei* in culture and in a mouse model.^3^

Acivicin binds to the glutaminase domain of CTPS and mimics the natural substrate l-glutamine (Figure 1). The enzyme is irreversibly inactivated through formation of a covalent adduct produced by nucleophilic attack by a Cys residue thiol group to C3 of the isoxazoline nucleus, with displacement of the chlorine atom.^5^ This mechanism of action indicates that the inhibitory activity should be influenced by the nature of the leaving group. In a previous work, we reported that 3-bromoacivicin (Figure 1) has a profile similar to that of acivicin in tests against a panel of tumor cell lines.^6^ However, a direct comparative study between acivicin and 3-bromoacivicin has not yet been made, and 3-bromoacivicin has only been previously evaluated as a racemate.

Herein we compare the CTPS inhibitory activity and the antitrypanosomal activity of acivicin with that of its 3-bromo analogue. We describe the development of a convenient synthesis of enantiomerically pure (α*S*,5*S*)-α-amino-3-bromo-4,5-dihydroisoxazol-5-yl acetic acid (3-bromoacivicin) allowing it to be tested, in parallel with acivicin, both for CTPS inhibition and as an antitrypanosomal agent.

## Results and Discussion

The synthesis of 3-bromoacivicin in a racemic form was previously described.^7^ However, a survey of the literature shows that an efficient preparation of enantiomerically pure (α*S*,5*S*)-3-bromoacivicin has never been described; the only reported methods require multistep procedures and rely on resolution of racemic mixtures through the formation of diastereomeric salts followed by crystallization.^8^

Herein we describe an efficient synthetic procedure to prepare (α*S*,5*S*)-3-bromoacivicin in a few synthetic steps with good overall yield. We took advantage of our previously described method for the preparation of (α*S*,5*S*)-acivicin.^9^ As detailed in references 9, the 1,3-dipolar cycloaddition of bromonitrile oxide to (*S*)-3-(*tert*-butoxycarbonyl)-2,2-dimethyl-4-vinyloxazolidine [(*S*)-**3**, Scheme 1], yields the two pairs of diastereomeric cycloadducts *erythro*-(+)-**4**/*threo-*(−)-**5** in a 65:35 ratio and in 88 % overall yield. The two isomers can be easily separated by silica gel column chromatography. We now used the *erythro* cycloadduct (+)-**4** as a key intermediate to prepare (α*S*,5*S*)-3-bromoacivicin. The acetonide function was removed upon treatment with an aqueous solution of acetic acid, and the *N*-Boc-protected amino alcohol (+)-**6** was oxidized to the corresponding carboxylic acid (+)-**7** by using ruthenium(IV) oxide and sodium periodate, followed by removal of the amino protecting group by treatment with a 33 % solution of hydrogen bromide in acetic acid. After cation-exchange chromatography, (α*S*,5*S*)-3-bromoacivicin (+)-**2** was obtained in its zwitterionic form. The overall yield after five steps starting from alkene (*S*)-**3** was 25 %. The starting material (*S*)-**3** can be purchased or easily prepared by starting from the commonly commercially available (*R*)-Garner’s aldehyde, following a published procedure.^10^

**Sheme 1:**
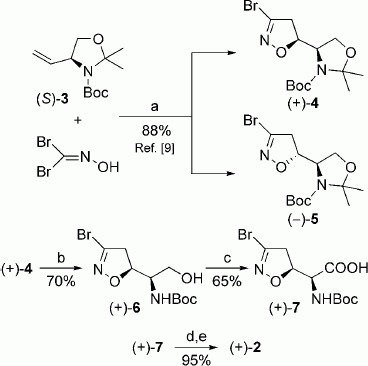
Reagents and conditions: a) NaHCO_3_/EtOAc, 24 h, RT; b) AcOH/H_2_O (5:1 *v*/*v*), 48 h, 40 °C; c) NaIO_4_, RuO_2_⋅H_2_O, H_2_O, CH_3_CN, CCl_4_, 2 h, RT; d) 33 % HBr/HAc, 5 min, RT; e) Amberlite IR-120H, 0.5 n NH_4_OH.

**Figure 2:**
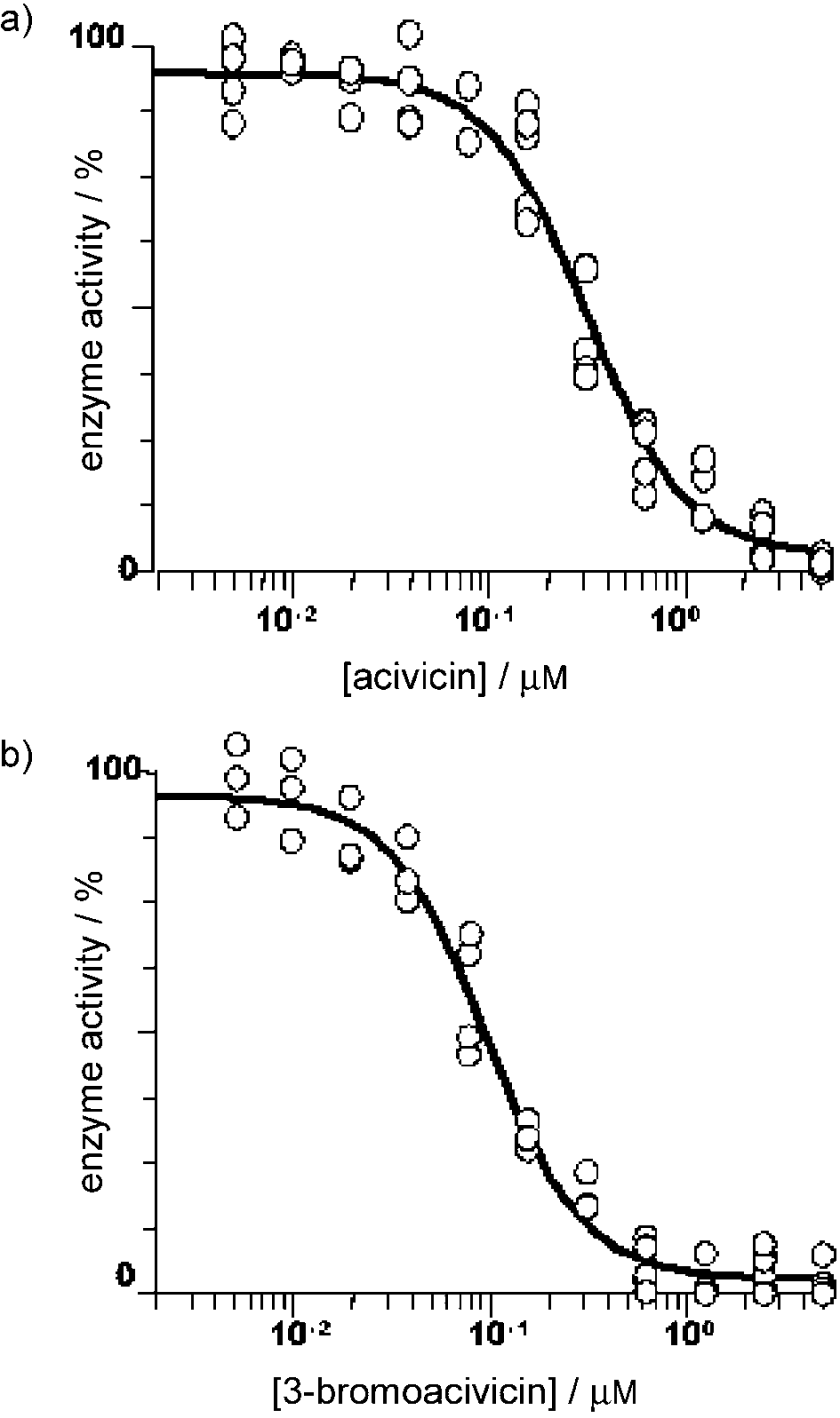
a) Acivicin and b) 3-bromoacivicin were assayed for inhibitory activity against purified recombinant *T. b. brucei* CTPS. Results are expressed as percentage of enzyme activity; the data were obtained from four replicate experiments (*n*=4), and IC_50_ values are expressed as the mean ±SD (acivicin IC_50_: 320±25 nM, 3-bromoacivicin IC_50_: 98±10 nM).

Acivicin (**1**) and 3-bromoacivicin (**2**) were assayed for inhibitory activity against purified recombinant *T. b. brucei* CTPS (Figure 2). Acivicin displayed an IC_50_ value of 320±25 nM, whereas 3-bromoacivicin was threefold more potent, with an IC_50_ value of 98±10 nM, indicating the nature of the leaving group plays a role in the interaction with the enzyme.

**Figure 3:**
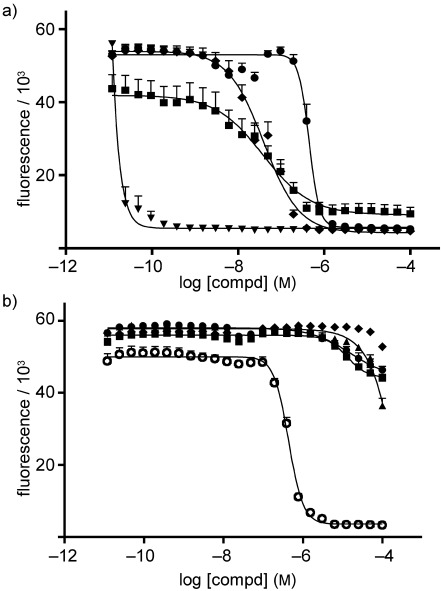
In vitro toxicity against a) *T. brucei* 427 and b) HEK mammalian cells. Wild-type trypanosomes and HEK cells were treated with acivicin (•), 3-bromoacivicin (▪), pentamidine (▾), diminazene (♦), and phenylarsine oxide (○) in 96-well plates at a range of concentrations as described in the Experimental Section. Alamar Blue was added 48 h post-treatment, and fluorescence was used as a measurement of parasite/cell viability. Results are expressed as the mean of each compound’s effective dose that kills 50 % of the cells (i.e., ED_50_) obtained from duplicates of three independent experiments (*n*=3); error bars represent standard errors.

We then evaluated the antitrypanosomal activity against the bloodstream form *T. brucei* (s427) (Figure 3 a). 3-Bromoacivicin displayed a 12-fold greater antitrypanosomal potency (ED_50_: 38±13 versus 450±10 nM), whilst the activities of 3-bromoacivicin and acivicin against mammalian HEK cells (Figure 3 b) were similar (ED_50_: 12.91±0.10 and 15.65±0.21 μM, respectively). Therefore, 3-bromoacivicin appeared to be a good drug candidate with antitrypanosomal activity in the low nanomolar range and a selectivity index of ∼300-fold relative to human HEK cells.

**Figure 4:**
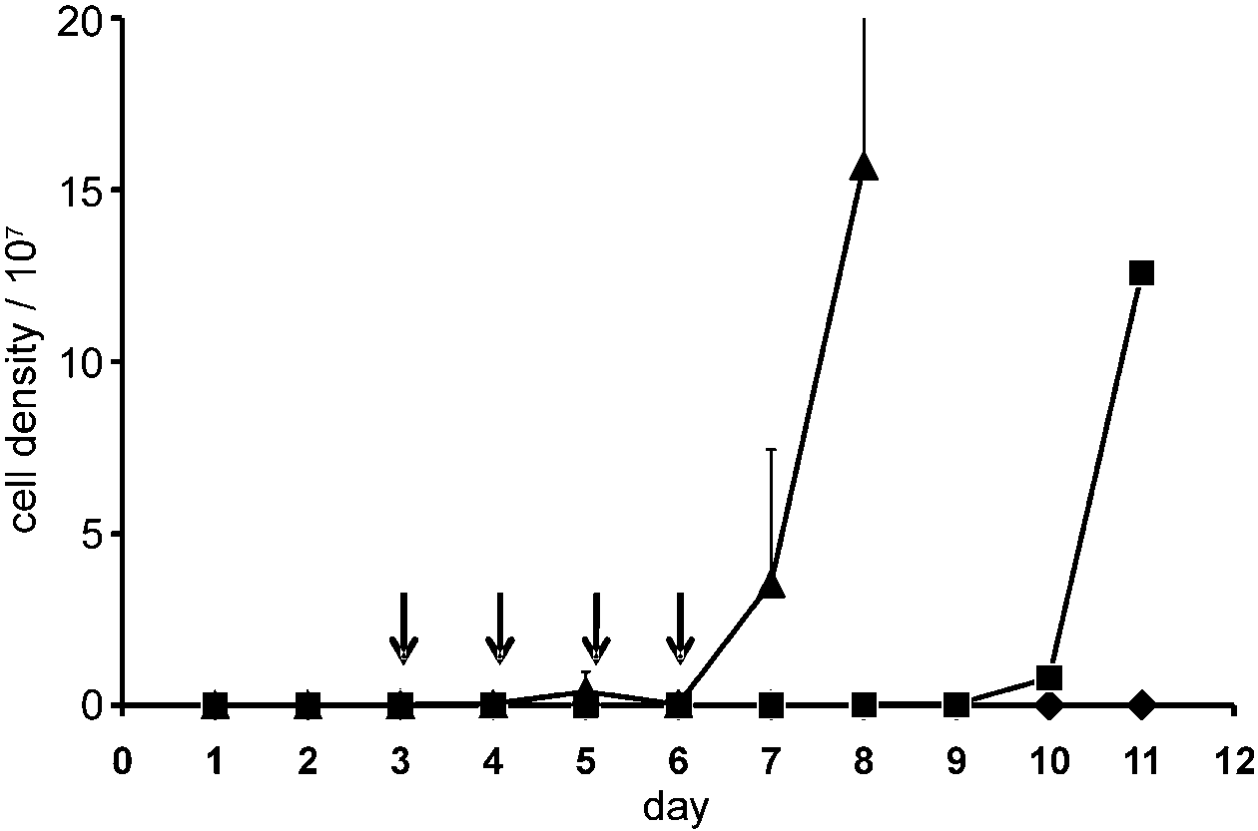
In vivo efficacy of 3-bromoacivicin against *T. brucei* 427 inoculated in adult female ICR mice (2×10^4^ parasites per mouse in 100 μL per i.p. injection). Mice in groups of three were treated daily for four days (represented by arrows “↓”) with 3-bromoacivicin 50 mg kg^−1^ (▪) and pentamidine 4 mg kg^−1^ (♦) given via i.p. injections (100 μL per injection). Untreated mice (▴) acted as negative control. Parasitemia levels were monitored daily by venepunctures, and for cases in which parasitemia reached, or was about to reach ∼10^8^ cells mL^−1^, the animal(s) were euthanized using Schedule 1, or at the end of procedure. Error bars represent standard deviations between the three animals within each group.

Given that pharmacokinetics and toxicity issues mitigated against the development of acivicin as a new trypanocide,3b we considered the superior activity of 3-bromoacivicin to offer the potential of lower dosing in mammals. We therefore evaluated the efficacy and primary toxicity profile of 3-bromoacivicin in vivo, using *T. b. brucei*-infected mice. Administration of a single dose of 3-bromoacivicin did not cause signs of acute toxicity (i.e., acute stress, difficulty in breathing, malaise, lack of appetite) at 10 and 50 mg kg^−1^. The individual animals did lose 9.4 and 11.9 % of their body weight at the respective doses of 10 and 50 mg kg^−1^ by day 14 post-treatment. However, this was deemed acceptable according to safety protocol measures, by which a compound is considered toxic if weight loss is >25 %. We then tested the ability of a 50 mg kg^−1^ dose to treat trypanosomes in the mouse model (Figure 4). Treatment of mice infected with *T. b. brucei* (s427) commenced on day 3, that is, two days post-infection, where the parasitemia level was at ∼2.5×10^5^ cells mL^−1^ for each animal. Parasites were not detectable throughout treatment days (i.e., days 3-6), indicating in vivo activity of the compound. However, parasites started to reappear on day 8, that is, two days after treatment was stopped, and proliferated progressively. The experiment had to be terminated on day 11 due to high parasitemia. Pentamidine as the positive control was effective at ensuring 100 % cure throughout the experiment (i.e., ‘0’ parasitic count). The parasitemia level peaked with the untreated group as expected, and these animals were culled on day 8 due to high parasitemia levels. This in vivo result with 3-bromoacicivin is similar to a previous observation for acivicin.3b Interestingly, in culture, acivicin and 3-bromoacivicin are trypanocidal after 72 h above 10 μM and at 1 μM after 96 h. However, they are only trypanostatic for 24 h at 100 nM; beyond this time point or below this concentration (10 nM), the trypanosomes continue to multiply, albeit at a decreased growth rate, depending on the concentration of inhibitor.

## Conclusions

In summary, we show that substitution of the 3-chloro group of acivicin with a bromo group to give 3-bromoacivicin increases the inhibitory potency against the target enzyme CTPS threefold. More interestingly, this translates into a 12-fold increase in the in vitro antitrypanosomal activity, while leaving the toxicity against mammalian cells unaffected. This could be due to an enhanced ability of the bromo analogue to enter trypanosomes, or due to its ability to inhibit targets in addition to CTPS in a manner that enhances its antitrypanosomal activity. Unfortunately, in vivo testing of 3-bromoacivicin at its maximum tolerable dose appeared to eradicate the trypanosomes initially, but was found to be ineffective at complete cure of the infection, as the parasites re-emerged upon removal of the treatment and proliferated progressively thereafter.

Future efforts will be devoted to the optimization of the lead compound 3-bromoacivicin through the design of new ligands with an increased inhibitory activity toward CTPS, which, according to the results presented herein, may lead to substantial increases in antitrypanosomal activity, while having minimal impact on the toxicity profile.

## Experimental Section

**Materials and methods.** All reagents were purchased from Sigma, and rodents were supplied by Harlan Laboratories UK Ltd. Acivicin was prepared according to a published procedure.9a Compound (+)-**4**^9^ was prepared according to published procedures by starting from (*S*)-**3**.^10^ ^1^H NMR and ^13^C NMR spectra were recorded with a Varian Mercury 300 (300 MHz) spectrometer. Chemical shifts (*δ*) are expressed in ppm and coupling constants (*J*) in Hz. MS analyses were performed on a Varian 320-MS triple quadrupole mass spectrometer with ESI source. Rotary power determinations were carried out using a Jasco P-1010 spectropolarimeter, coupled with a Haake N3-B thermostat. TLC analyses were performed on commercial silica gel 60 F_254_ aluminum sheets; spots were further evidenced by spraying with a dilute alkaline solution of KMnO_4_ or with ninhydrin. Melting points were determined on a model B 540 Büchi apparatus and are uncorrected.

***tert*****-Butyl-(*R*)-1-[(*S*)-3-bromo-4,5-dihydroisoxazol-5-yl]-2-hydroxyethylcarbamate (+)-6.** Compound (+)-**4** (1.3 g, 3.7 mmol) was treated with a 5:1 mixture of AcOH/H_2_O (30 mL). The solution was stirred at 40 °C for 48 h. The solvent was evaporated, the residue was dissolved in EtOAc (20 mL) and washed with H_2_O (2×10 mL). The organic phase was dried over anhydrous Na_2_SO_4_ and the solvent was evaporated under reduced pressure. The crude material was purified by column chromatography (cyclohexane/EtOAc 7:3) to give compound (+)-**6** (0.80 g, 70 % yield), which was crystallized from *i*Pr_2_O as colorless needles; mp: 111–113 °C; *R*_f_=0.31 (cyclohexane/EtOAc 7:3); [*α*]_D_=+92.6 (*c*=1.03, CHCl_3_); ^1^H NMR (CDCl_3_): *δ*=1.45 (s, 9 H), 1.89 (br s, 1 H), 3.25–3.38 (m, 2 H), 3.67–3.82 (m, 2 H), 3.88–3.99 (m, 1 H), 4.77 (ddd, *J*=8.7, 8.7, 8.7, 1 H), 5.11 (br s, 1 H); ^13^C NMR (CDCl_3_): *δ*=28.5, 44.6, 54.2, 61.4, 80.5, 81.0, 138.5, 156.2; MS [*M*+H]^+^: 309.2; Anal. calcd for C_10_H_17_BrN_2_O_4_: C 38.85, H 5.54, N 9.06, found: C 38.98, H 5.68, N 9.20.

**(α*S*,5*S*)-α-*tert*-Butoxycarbonylamino-3-bromo-4,5-dihydroisoxazol-5-yl acetic acid (+)-7.** Compound (+)-**6** (0.80 g, 2.6 mmol) was dissolved in a 10:7:7 mixture of H_2_O, CH_3_CN and CCl_4_ (24 mL). NaIO_4_ (2.22 g, 10.4 mmol) and a catalytic amount of RuO_2_⋅H_2_O (6.6 mg, 0.05 mmol) were added and the suspension was vigorously stirred at room temperature for 2 h. After disappearance of the starting material, CH_2_Cl_2_ (50 mL) and H_2_O (50 mL) were added. The organic phase was separated and evaporated under reduced pressure. The residue was dissolved in EtOAc (10 mL), the organic layer was extracted with a 10 % aqueous solution of K_2_CO_3_ (3×10 mL) and the aqueous phase was made acidic with 2 n HCl and newly extracted with EtOAc (3×10 mL). The organic extracts were washed with brine, dried over anhydrous Na_2_SO_4_, and the solvent evaporated to give (+)-**7** as a white foam (0.55 g, 65 % yield); [*α*]_D_=+170.7 (*c*=0.5, CHCl_3_); ^1^H NMR (CDCl_3_): *δ*=1.45 (s, 9 H), 3.30–3.60 (m, 2 H), 4.52 (dd, *J*=3.9, 8.0, 1 H), 5.00 (ddd, *J*=3.9, 7.7, 11.0, 1 H), 5.45 (bs, 1 H); ^13^C NMR (CDCl_3_): *δ*=28.5, 44.3, 56.3, 81.5, 82.1, 138.6, 155.7, 172.2; MS [*M*−H]^+^: 320.7.

**(α*S*,5*S*)-α-Amino-3-bromo-4,5-dihydroisoxazol-5-yl acetic acid (+)-2.** Compound (+)-**7** (0.55 g, 1.7 mmol) was treated with a 33 % solution of HBr in AcOH (20 mL) at room temperature for 5 min. The volatiles were removed under vacuum, the residue was dissolved in H_2_O and submitted to cation-exchange chromatography using Amberlite IR-120H. The acidic solution was slowly eluted onto the resin, and then the column was washed with H_2_O until the pH was neutral. The compound was eluted off the resin with 0.5 n aqueous ammonia, and the product-containing fractions (detected with ninhydrin stain on a TLC plate) were combined. The solvent was freeze-dried to give (+)-**2** as a white powder (0.36 g, 95 % yield). Colorless prisms from MeOH/H_2_O; mp: >175 °C (dec.); [*α*]_D_=+178.2 (*c*=0.10, H_2_O); ^1^H NMR (D_2_O): *δ*=3.35 (dd, *J*=8.2, 17.1, 1 H), 3.45 (dd, *J*=11.0, 17.1, 1 H), 3.92 (d, *J*=3.3, 1 H), 5.08 (ddd, *J*=3.3, 8.2, 11.0, 1 H); ^13^C NMR (D_2_O): *δ*=42.9, 56.1, 79.7, 140.9, 170.0; MS [*M*+H^+^]: 223.0; Anal. calcd for C_5_H_7_BrN_2_O_3_: C 26.93, H 3.16, N 12.56, found: C 27.00, H 3.19, N 12.49.

**CTPS enzyme assays.** *T. brucei* CTPS was recombinantly expressed and purified as described elsewhere.3c A coupled spectrophotometric assay was used consisting of a final volume of 150 μL containing: 70 mM MOPS pH 7.6, 150 mM KCl, 12 mM MgCl_2_, 500 μM ATP, 1.0 mM DTT, 1 mM phosphoenolpyruvate, 0.5 mM NADH, 1.5 units of pre-mixed pyruvate kinase and lactate dehydrogenase, 655 μM GTP, 625 μM UTP, 1.25 mm l-Gln, and 13 μg purified *T. brucei* CTPS.3c Various concentrations of acivicin and 3-bromoacivicin were pre-incubated with *T. brucei* CTPS while on ice for 5 min, prior to assaying.

**In vitro toxicity assays for** ***T. brucei*** **(s427) and HEK mammalian cells.** *T. brucei* (s427) was cultured to the optimum density of 1–2×10^6^ cells mL^−1^ in HMI-9 supplemented with 10 % fetal calf serum (FCS) under environmental conditions of 37 °C and 5 % CO_2_. Solutions of test compounds were prepared in culture media at stock concentration of 200 μM and diluted serially (1:2) across the 96-well, flat-bottom solid white plates to give a total of 11 decreasing concentrations (100 μL per well). The last well of each series was left blank (drug free, negative control). Cells were prepared at the concentration of 4×10^4^ cells mL^−1^ and added to each well of the respective compound series (100 μL well^−1^). Plates were incubated at 37 °C and 5 % CO_2_ for 48 h prior to the addition of a solution of Alamar Blue (20 μL per well, 0.49 mM in 1×PBS, pH 7.4) followed by a further 24 h. Assay end points were measured fluorimetrically with a fluorescence spectrometer (FluoStar, BMG LabTech, Germany) and Optima program set at *λ*_excitation_=544 nm and *λ*_emission_=590 nm. Data were analyzed using Prism 5.0 software to obtain ED_50_ values. Experiments were performed in duplicate and repeated three times. A similar Alamar Blue assay was carried out with human embryonic kidney (HEK) cells, cultured in DMEM supplemented with 10 % FCS and 2 mm l-Gln. HEK cells were plated at an initial concentration of 3×10^5^ cells mL^−1^ (100 μL per well) and incubated with test compounds for 16 h prior to the addition of Alamar Blue solution.

**Trypanocidal or trypanostatic effects of acivicin and 3-bromoacivicin.** Cultured bloodstream *T. brucei* parasites (1×10^5^ cells mL^−1^) were incubated in replicates of six with various concentrations (1 nM, 10 nM, 100 nM, 1 μM and 10 μM) of either acivicin or 3-bromoacivicin. The parasites were counted every 24 h. At 72 and 96 h, triplicate samples of the cells treated with 1 and 10 μM acivicin or 3-bromoacivicin were centrifuged (500 *g*, 5 min), washed with fresh HMI-9 medium, and suspended in fresh medium in the absence of acivicin or 3-bromoacivicin and monitored for a further 10 days.

**In vivo acute toxicity and efficacy of 3-bromoacivicin.** Female adult ICR mice were tested with 3-bromoacivicin at progressive increasing doses (10 and 50 mg kg^−1^) for signs of acute toxicity. Compound was dissolved in H_2_O and administered via intraperitoneal (i.p.) injection (100 μL per dose). The treated rodents were observed and weighed daily for 14 days. Euthanasia via Schedule 1 methods (see below) were carried out at signs of acute toxicity and/or body weight loss of >25 % or at the end of procedure. In the efficacy assay, mice were infected with *T. brucei* (s427) (2×10^4^ parasites per mouse, 100 μL i.p. per injection) in groups of three. Once infection was established (normally 48 h post-infection), mice were treated with pentamidine (4 mg kg^−1^, 100 μL i.p. per injection, positive control) and 3-bromoacivicin (50 mg kg^−1^, 100 μL i.p. per injection). Untreated mice acted as negative control. Treatments were prepared fresh daily and repeated daily for a further 3 days (i.e., four doses in total). Daily monitoring of parasitemia levels was carried out via venepuncture. For instances in which the parasitemia level reached or was about to reach ∼10^8^ cells mL^−1^, or at the end of the procedure, mice were euthanized using Schedule 1 methods according to legislation of the United Kingdom Animals (Scientific Procedures) Act 1986, the national and the University of Glasgow maintenance and care guidelines.
